# Compositional and functional variability of the gut microbiome in children with infantile colic

**DOI:** 10.1038/s41598-023-36641-z

**Published:** 2023-06-12

**Authors:** Samat Kozhakhmetov, Zarina Meiirmanova, Nurislam Mukhanbetzhanov, Zharkyn Jarmukhanov, Elizaveta Vinogradova, Shamil Mureyev, Saniya Kozhakhmetova, Marina Morenko, Kseniya Shnaider, Arailym Duisbayeva, Almagul Kushugulova

**Affiliations:** 1 Laboratory of Microbiome, Center for Life Sciences, National Laboratory Astana, Nazarbayev University, 53 Kabanbay batyr ave., Block S1, Nur-Sultan, Z05H0P9 Republic of Kazakhstan; 2 Department of Children’s Diseases with Courses in Allergology, Hematology and Endocrinology, NJSC “Astana Medical University”, Astana, Z01G6C5 Kazakhstan; 3 National Center for Biotechnology, Astana, Z05K8D5 Kazakhstan

**Keywords:** Bacteria, Dysbiosis

## Abstract

The inconsolable crying of a child for no apparent reason at an early age is a source of excitement and anxiety for parents. Previous studies have reported that crying may be caused by discomfort associated with the occupation of the intestines of the newborn by microbiota and its vital activity. We conducted a prospective observational study in which 62 newborns and their mothers were recruited. The study comprised two groups, each consisting of 15 infants with colic and 21 controls. Colic and control groups were vaginally born and exclusively breastfed. Fecal samples from children were collected over time from day 1 to 12 months. Full metagenomic sequencing of fecal samples from children and their mothers was carried out. It was determined that the trajectory of the development of the intestinal microbiome of children with colic was different from the group without colic. In the colic group, a depleted relative abundance of *Bifidobacterium* and enrichment of *Bacteroides Clostridiales* was found, while the microbial biodiversity in this group was enriched. Metabolic pathway profiling showed that the non-colic group was enriched by amino acid biosynthetic pathways, while the feces microbiome of the colic group was enriched by glycolysis metabolic pathways that correlated with the *Bacteroides* taxon. This study shows that infantile colic has a definite relationship with the microbiome structure of infants.

## Introduction

Infantile colic is a common condition and has no generally accepted etiology. The condition is characterized by inconsolable crying and screaming for no apparent reason. The child has a tense stomach, tightened legs, and a red face. Gastrointestinal disorders, psychosocial, hormonal, nutrition, etc. are discussed as suggested causes^1,2^. The prevalence of this condition ranges from 8 to 40% according to the results of various studies^3^. One of the main assumptions in breastfeeding is gastrointestinal disturbances in response to substances in the mother’s diet^4^.

Early childhood is critical for a child’s development and influences the process of microbiome formation and lifelong health^5^. From the moment of birth, the infant's microbiota begins to develop intensively, acquiring a starting set of microbiota from the mother and environmental factors. The process of development of the gut microbiome is a key step in the formation of a healthy immune defense of the body and a failure in its adequate development can lead to long-term undesirable effects in the future^6^. Interestingly, the development of the intestinal microbial community can follow different scenarios. Especially when you consider that the intestinal microbiota in early childhood is rapidly evolving toward bacterial composition and diversity. Research in the past decade suggests a link between the trajectory of the gut microbiome in young children and infantile colic. So there is evidence showing a significantly increased relative abundance of H_2_-producing bacteria in the gut microbiome of children with colic^7^. Several taxa have been identified that are significantly associated with colic: *Acinetobacter, Lactobacillus iners*^8^, *Clostridium, Lactobacillus*, and *Klebsiella*^9^. Also, the colic phenotype was positively correlated with the content of *Serratia, Vibrio*, and *Pseudomonas*^10^. A decrease in the relative content of bifidobacteria by an average of 30 times was observed^8^. In addition, an association of infantile colic in infants with formula feeding and *Escherichia coli* was found^11^. Another fact that strongly indicates the association of the intestinal microbiome with the occurrence of infantile colic is the ability of the probiotic strain *Lactobacillus reuteri* DSM 17938 to significantly improve the symptoms of infantile colic.

Recent studies have suggested that the gut microbiome of infants with infantile colic may be influenced by both the microorganisms present in the meconium and inherited microorganisms passed on from the mother to the infant during birth and breastfeeding^12,13^. Also, some studies have found that infants with colic have a different composition of microorganisms in their meconium compared to infants without colic. These differences may be related to the mode of delivery, with infants born via cesarean section having a less diverse meconium microbiome than those born vaginally^14^.

In several studies, it was found that infants with colic had higher levels of specific microorganisms, such as *Veillonella ratti, Anaerobutyricum hallii* (*Eubacterium hallii*)^7^ and *Roseburia*, that are known to produce gas in the intestines. These microorganisms ferment carbohydrates in the gut and produce gases like hydrogen, carbon dioxide, and methane. This can lead to an increase in intestinal gas and discomfort, which may contribute to colic symptoms. However, Mai et al.^2^ showed that the level of actinobacteria (95% of which are bifidobacteria) is significantly lower in the group of children with infantile colic compared to the group without colic. Another study found that the taxa *Bacteroides, Ruminococcus, Roseburia, Clostridium, Eubacterium, Desulfovibrio*, and *Methanobrevibacter*, which produce gas and other substances that can lead to inflammation in the gut, were increased in infants with colic^15^. This work aims to investigate the structural and metabolic variability of the gut microbiome in children with infantile colic.

## Materials and methods

### Ethics statement

The study was approved by the local ethics committee of the National Laboratory Astana Nazarbayev University, IORG0006963, Protocol No. 05-2020, Astana, Kazakhstan. Respondents were informed about the aims of the study and signed an informed consent form. Interaction with respondents was carried out in accordance with the general instructions and rules.

### Study design, recruitment and sample collection

This prospective cohort observational study included 62 newborns born from April 17, 2021, to April 17, 2022, at Perinatal center no. 2, Astana city (Kazakhstan). Written informed consent was obtained from volunteers who expressed a desire to participate in the study for themselves and their children. The study did not include children born with any pathology and premature babies. Neonatal meconium samples were collected by pre-trained personnel. Subsequently, samples were collected at collection points 1, 3, 6, and 12 months after birth through family visits by research pediatricians. During the visit, researchers measured weight, height, method of feeding the child, taking over-the-counter drugs, the timing of formula administration, and complementary foods. A child who once received infant formula was considered to be on mixed feeding. Those children who never received breastfeeding were considered formula-fed. The follow-up collection in this study did not include children taking antimicrobials. The study group was divided into subgroups according to the occurrence of infantile colic. Infantile colic was assessed according to the following Rome IV committee criteria: (a) an infant who is less than 5 months of age when the symptoms start and stop; (b) recurrent and prolonged periods of infant crying, fussing, or irritability reported by caregivers that occur without obvious cause and cannot be prevented or resolved by caregivers; (c) no evidence of infant failure to thrive, fever, or illness; (d) Caregiver reports infant has cried or fussed for 3 or more hours/day during 3 or more days in 7 days in a telephone or face-to-face screening interview with a researcher or clinician; (e) total 24-h crying plus fussing in the selected group of infants is confirmed to be 3 h or more when measured by at least one, prospectively kept, 24-h behavior diary^16^. Children were enrolled in the colic subgroup based on the parent's report of colic using the above criteria.

### Sample preparation, processing, and sequencing

Samples of meconium and feces were collected immediately after defecation from the diaper in a DNA/RNA Shield Collection Tube (Zymo Research, R1101). Fecal samples were stored in a refrigerator at + 4 °C until DNA extraction. Genomic DNA from fecal samples was extracted using the ZymoBIOMICS DNA Miniprep Kit (Zymo Research, D4300), and sterile µQ water was used as a negative extraction control. A qualitative control of DNA isolation was performed by OD260/280 Nanodrop and electrophoresis in a 1% agarose gel. The concentration and purity of each DNA sample were determined using an Invitrogen Qubit 3.0 Fluorimeter (Invitrogen, Carlsbad, California, United States). Sterile µQ water served as a negative control. Sequencing was performed on the Illumina NovaSeq 6000 platform at the laboratory of Novogene (Beijing, China) following the standard Illumina protocols.

### Data processing and statistics

When analyzing the obtained raw sequencing data, we used a set of integrated methods of taxonomic, deformation, functional, and phylogenetic profiling of metagenomes bioBakery 3^16^. MetaPhlAn 4 was employed for taxonomic profiling^17^, while HUMAnN 3 utilized native UniRef90 annotations for the functional profiling of genes, pathways, and modules from metagenomes^16^. The recommended parameters provided by the developers were applied for all tools used in the analysis. Chi-square and t-test Shapiro were used to compare groups of infants at baseline.

Within-sample community (α-diversity) was assessed using Shannon and Simpson indices, observed and estimated number of taxa (Observed and Chao1 index correspondingly), and compared between groups using a Mann–Whitney U test. Divergence in community composition between samples (β-diversity) was assessed by calculating a Bray–Curtis (abundance) and a Jaccard (presence/absence) distance metric. Ordination was visualized using Principal Coordinate Analysis (PCoA) and compared using the ANOSIM and PERMANOVA tests with 999 permutations on Hellinger-transformed data. Diversity calculation, ordination, ANOSIM and PERMANOVA tests were performed in Python 3 using the “scikit-bio 0.5.6” package. Pathway ordination was performed using principal coordinate analysis (PCA) on centered log ratio (CLR) transformed data in Python 3 using the scikit-learn 1.2.0 package with default parameters. Features with high absolute impact on PCA ordination and varying orientation were selected for visualization. In addition, ordination of functional variables based on their importance in discriminating between groups was performed using a random forest algorithm using the “scikit-learn 1.2.0” package. Ordination was calculated by calculating the effect of the feature on performance using the permutation test (with 30 repeats) during nested five-fold stratified cross-validation in the inner loop. The leave-one-out strategy in the outer loop was used for the overall calculation of the discriminative power in terms of the area under the curve (AUC). Hyperparameter tuning was performed in a nested loop during cross-validation. STAMP 2.1.3 software was used to identify significant functional characteristics between experimental groups using default parameters for 2-group comparison and confidence interval (CI) calculation. The effect size threshold was set at 0.1. LEfSe was used to identify the most differentially distributed taxa and functional features with significantly large effect sizes. The default LDA score threshold of 2 and the default significance threshold of 0.05 were used in the LEfSe analysis. Correlation analysis of functional and taxonomic features was performed using Kendall’s coefficient for only significantly differentially abundant features in Python 3 using “SciPy 1.7.0”. Visualization was performed using the “matplotlib 3.7.0” library.

### Ethics declarations

The study was conducted according to the guidelines of the Declaration of Helsinki, and approved by the local ethics committee of the National Laboratory Astana Nazarbayev University IORG0006963, (Protocol No. 05-2020).

### Informed consent

Written informed consent was obtained from all study participants or their legal guardians.

## Results

### Subjects of the study, characteristics

To analyze the microbiome in early life and the ability to change, we analyzed the fecal microbiota of a cohort of healthy Kazakh infants born in 2021 with an average Apgar score of 8/9. Fecal samples were collected following the same procedure at five-time points, 3 DB—meconium (12–48 h), feces at 1 (1 MB), 3 (3 MB), 6 (6 MB), and 12 months (12 MB). We recruited 62 children and their mothers into the general study group. Of the total group, 50 children were delivered vaginally and 11 by cesarean section. 15 children were on the formula and mixed feeding. After 1 month of life in relation to infantile colic, 15 children with colic and 21 children from the cohort were selected, who were exclusively breastfed and were born vaginally (Table 1). We observed the cessation of colic in children by 3 months. All children were born after a normal pregnancy. After diagnosing infantile colic, infants in both groups did not show significant differences. As seen from Table 1, physiological indicators demonstrate similarity. The study included 144 fecal samples, including 34 meconium samples, 1-month feces—36 samples, 3 months—29 samples, 6 months—32 samples, and 12 months—13 samples.

**Table 1:** Baseline characteristic of infants.

Variables	Colic	Non-colic	p-value
N (male/female)	15 (6/9)	21 (12/9)	0.49896
Gestational (days)	280.4 ± 5.8 (269–289)	278.6 ± 9.0 (254–290)	0.49443
Birth length, cm	54 ± 2.4 (50–58)	54.9 ± 2.2 (52–59)	0.25774
Birth weight, g	3517 ± 492 (2850–4425)	3720.5 ± 544 (3030–5060)	0.25867
Breastfeeding, n (%)	All	All	–
Apgar score (A/S)	8/9	8/9	–

Values are means ± SD (range) or n.

Most birth samples (collected within the first 12–24 h) had low DNA concentrations and were excluded from subsequent analysis.

The study found that the average age of the mothers was 30.1 ± 5.2 years, and the average birth weight of children in the sample was 3.69 ± 0.49 kg. The mean gestational age was 281.5 ± 8.5 days (with a range of 259–304 days) for all newborns cohort. Detailed characteristics of both the colic and non-colic groups are provided in Table 1. However, due to some parents refusing to participate in the study, we were unable to collect samples from all of the study participants at each time point.

### Compositional variability of the early life gut microbiota

**Figure 1:**
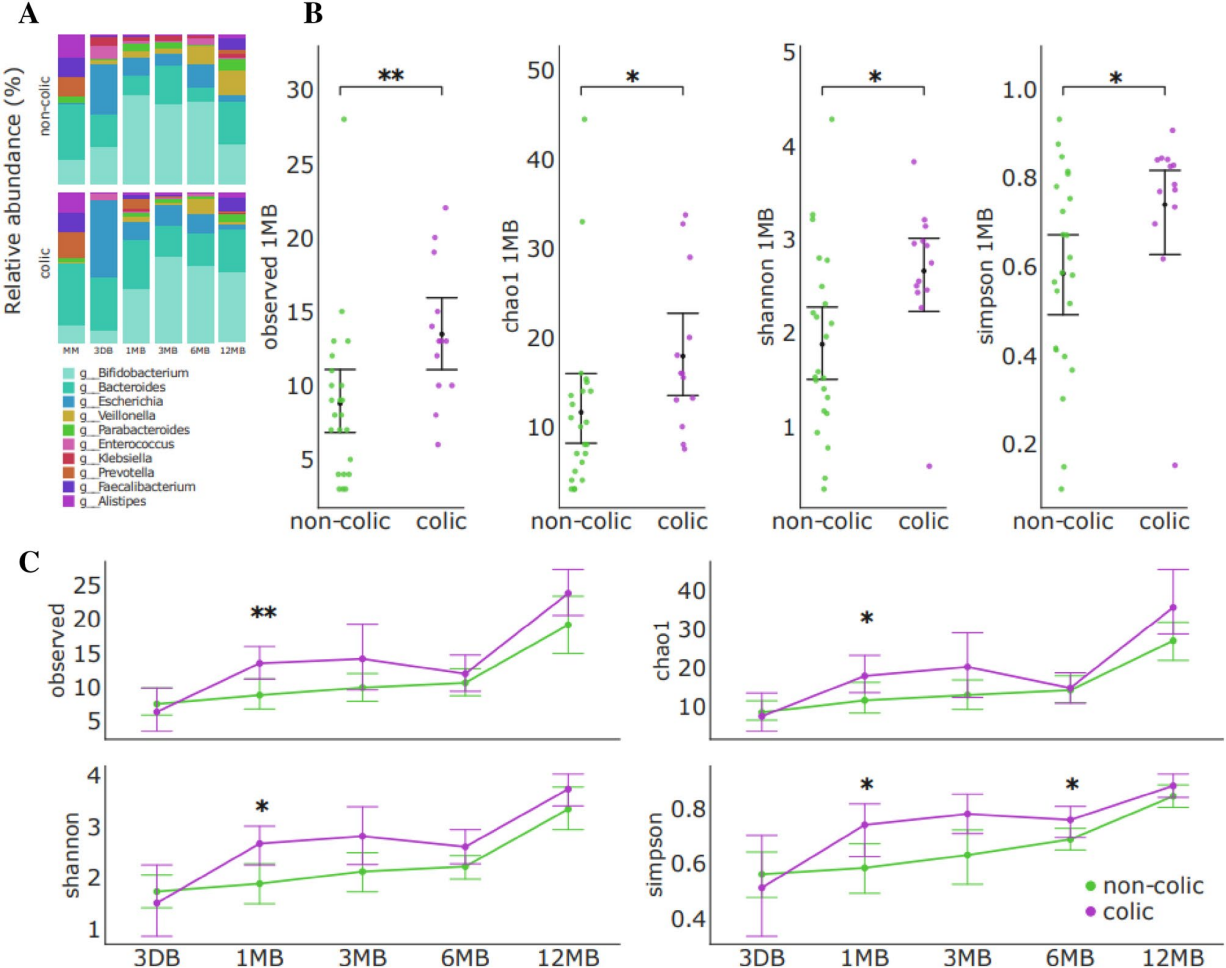
Composition and dynamics of the fecal microbial community in early life up to 12 months. (**A**) Mean relative abundance of taxa at sampling points (3 DB—1 day, 1 MB—1 month, 3 MB—3 months, 6 MB—6 months, 12 MB—12 months and MM—maternal microbiome). (**B**) Alpha biodiversity across the entire cohort and rates with and without infantile colic after 1 month of age. (**C**) Dynamics of microbiota development by groups: red line—all groups; green line—non-colic group; purple line—colic group, whereas the whiskers represent the 95% confidence interval (CI) (with 2000 bootstrap resampling); Mann–Whitney U test, *p ≤ 0.05, **p ≤ 0.01.

In order to investigate the variations in microbial composition during early life, we conducted an analysis of the fecal microbiota using shotgun sequencing. On average, each paired-end (PE) sequencing sample generated 3.6 GB of raw data with a Q30 score of 91.75%. The computational tool MetaPhlAn 4 was employed for microbial community profiling and processed an average of approximately 44 million reads for each sample. The findings from this analysis revealed that *Bifidobacterium, Bacteroides*, and *Escherichia* are the predominant components of the fecal microbiota in early infancy. These results are illustrated at the genus level in Fig. 1A.

**Figure 2:**
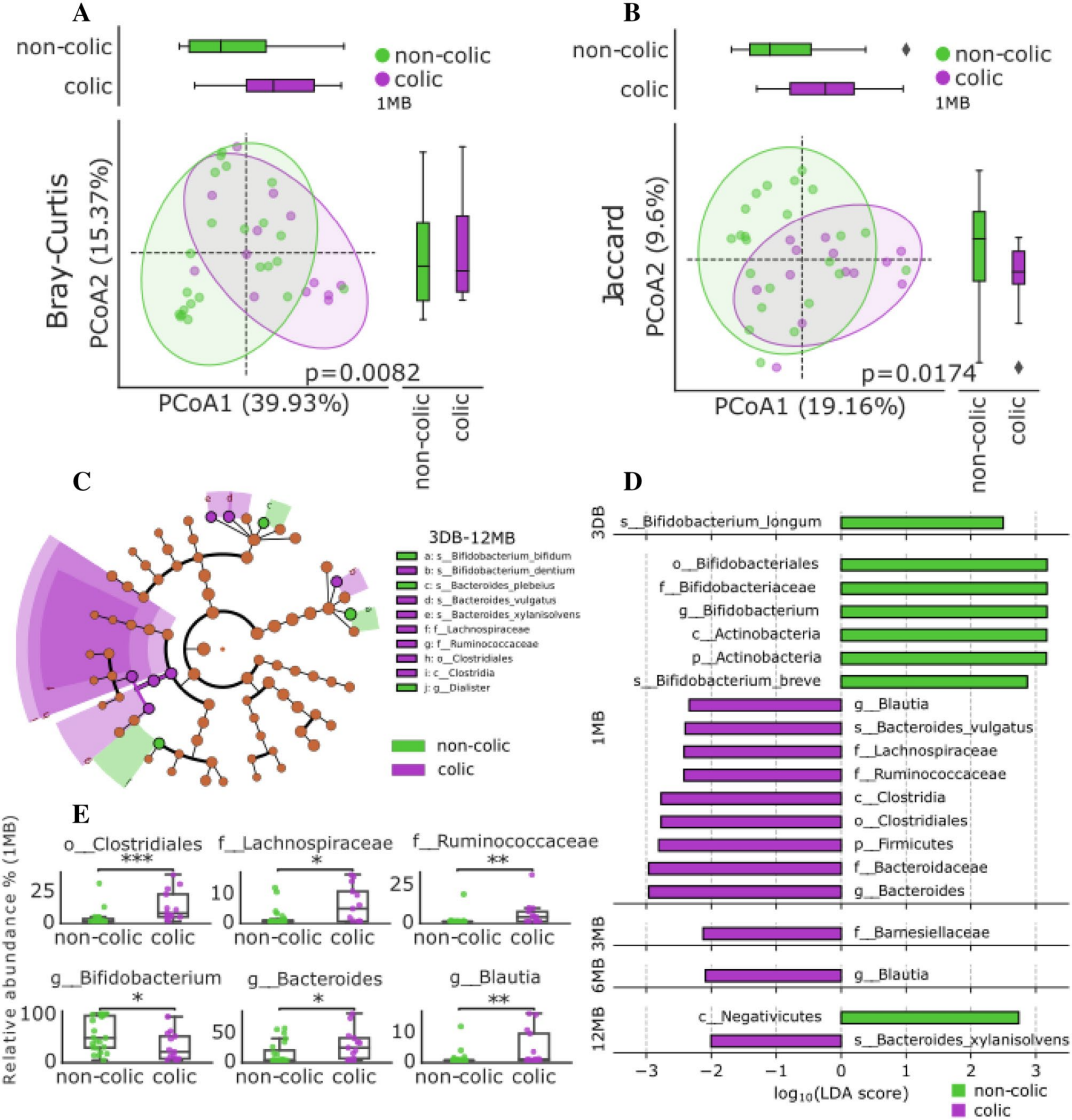
Composition of the bacterial community in the intestines of children in early life (1 month) with and without infantile colic. PCoA ordination plot of β-diversity of fecal microbiota in infants with colic and non-colic based on Bray–Curtis dissimilarity (**A**) and Jaccard distance (**B**). (**C**) The cladogram generated by LEfSe shows the differences in taxa between infantile colic and non-colic groups in samples across all collection points. The central dot on the cladogram represents the kingdom; each subsequent circle is phylogenetically one step lower (phylum, class, order, family, genus, species). Areas in green represent taxa that were enriched in the non-colic group, purple are areas enriched in the infantile colic group. (**D**) The linear discriminant analysis (LDA) effect size (LEfSe plot) p < 0.05. An LDA score (log 10) > 2 indicates a significantly different enrichment of bacteria taxa in the colic group (purple) compared to the non-colic group (green). p, phylum; c, class; o, order; f, family; g, genus; s, species. (**E**) Bacterial species over-depleted/represented in colic group compared to non-colic group; Mann–Whitney U test, FDR-corrected, *p ≤ 0.05, **p ≤ 0.01, ***p ≤ 0.001.

The results demonstrate high intergroup taxonomic differences in the composition of the microbiome of the colic and non-colic groups. Determining the mean differences in the Shannon Biodiversity Index clearly demonstrated an increase in biodiversity and abundance over the entire sample over time, as well as an accelerated enrichment of the gut microbiome in the group of children with colic compared to the gut microbiota without colic. Interestingly, in the group with colic, bacterial species diversity, after 1 month of life, is higher compared to the group without infantile colic (Fig. 1B), which is demonstrated by the observed and estimated number of taxa (Observed (p ≤ 0.01) and Chao1 index (p ≤ 0.05), correspondingly), Shannon index (p ≤ 0.05) and Simpson index p ≤ 0.05). By the age of 6 months, we observed a decrease in biodiversity, which coincided with a reduction in breastfeeding and the introduction of complementary foods and solid foods into the children’s diet. The group average diversity decreased due to the following taxa at the genus level: *Akkermansia, Enorma, Roseburia, Ruminococcus, Oscillibacter, Lachnospiraceae_unclassified, Coprococcus, Desulfovibrionaceae_unclassified, Lachnospira, Pseudomonas, Raoultella, Hungatella*. However, the exact trigger for this process is still unknown. At the same time, anatomical and physiological development, as well as the development of local immunity in the intestinal mucosa of the child, may play a role in this process. However, after the first 12 months of life, alpha biodiversity indices show similar values for the colic group and non-colic group. Also, Fig. 1C, Supplement Fig. 1, shows a dramatic increase in biodiversity from 6 to 12 months of age, which is likely due to an increase in the dietary menu, the elimination of breast milk in the study group of children, and the introduction of solid foods. The determination of the top 10 most represented taxa of bacteria showed that the main genera are, in descending order: *Bifidobacterium, Bacteroides, Escherichia, Veillonella, Parabacteroides, Enterococcus, Klebsiella, Prevotella, Faecalibacterium, Alistipes*. The results showed that the average relative frequency of occurrence of bifidobacteria in the feces of infants in the group with infantile colic (28.65% average relative abundance) after 1 month of life was ~ 1.95 times lower (MW, FDR corrected p = 0.023) compared to the group of children without colic (54.84% average relative abundance) (Fig. 2E). We also observed reduced abundance of *Parabacteroides, Enterococcus*, and *Klebsiella*. In contrast, there is an increased relative abundance of bacteria of the genus *Bacteroides, Prevotella, Faecalibacterium*, and *Alistipes* in colic group (Fig. 1A). We also observed an altered composition of the microbiota in both maternal feces and infant meconium (Fig. 1A). If the ratio of the average relative abundance of the bacterial genus in the two groups was approximately the same for mothers, then the bacterial composition of meconium between groups of newborns had a very high difference. Thus, the group of children with infantile colic had a lower representation of *Bifidobacterium, Enterococcus*, and *Klebsiella*, in contrast to *Bacteroides* and *Escherichia* dominated.

PCoA ordination based on dissimilarity (Bray–Curtis) and presence/absence distance (Jaccard) on hellinger-transformed data at the species level revealed group-dependent segregation between a group of infants with and without infantile colic in snapshot 1 MB (Bray–Curtis, PERMANOVA p = 0.0082, Pseudo-F = 3.59221) and (Jaccard, PERMANOVA p = 0.0174, Pseudo-F = 1.856741). Linear discriminant analysis (LDA) combined with effect size measurements (LEfSe) and derived cladogram (Fig. 2C, D) showing the significantly abundant bacterial taxa belonging to the phylum *Firmicutes*, class *Clostridia*, order *Clostridiales*, families *Ruminococcaceae*, genus *Blautia* in a group of samples from children with infantile colic, while genus *Blautia* was also predominant after 6 months of early life, and the species Bifidobacterium bifidum, in contrast, in time point 1 MB, was soloist in the group of samples from children without colic. It is known that in early life, the predominant taxa in the “normal” development of the intestinal microbial flora are *Bifidobacterium* species, which, through breast milk oligosaccharide (HMO)-utilizing, acidify the intestinal environment, forming acetate and lactate and other metabolites, thereby inhibiting the growth of potentially pathogenic proteolytic microorganisms. Prevalence of taxa belonging to the class *Clostridia* (about 12% of the average relative abundance in colic versus 3% in the group without colic) may indicate excessive maturation and diversification of the gut microbial composition in the colic group (Fig. 2E).

### Differences in metabolic pathways between the colic and non-colic group

**Figure 3:**
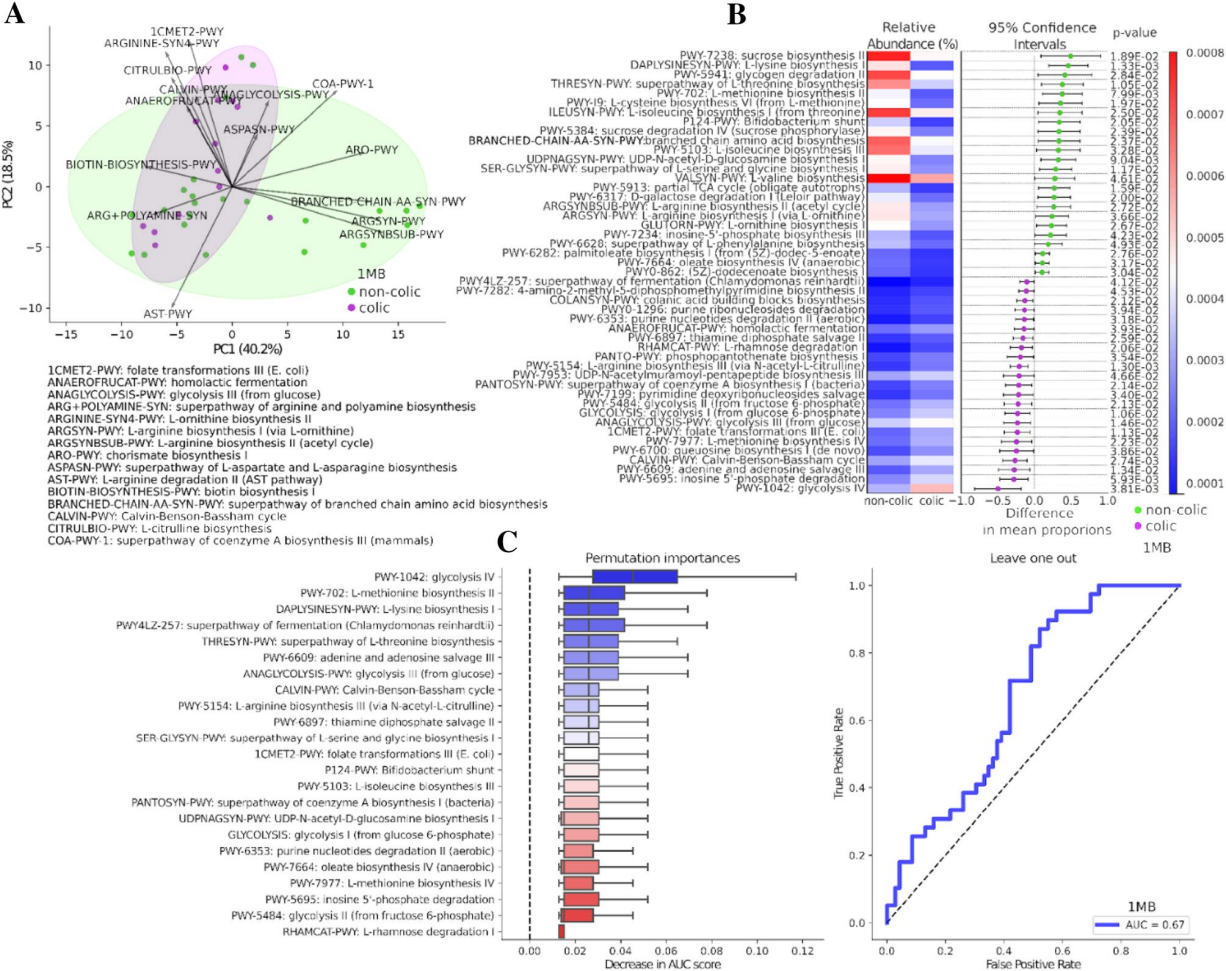
Gut microbiome metabolic pathway infants after 1 month of life. (**A**) Principal component analysis (PCA) ordination of metabolic pathways in the colic and non-colic groups based on clr-transformed (center log ratio transformation) functional abundance data. Top impacting ordination pathways of different orientation (to avoid overlap) for two groups are shown as arrows in the direction of impact. (**B**) Extended error bar plots showing functional properties that differ between non-colic and colic infant gut microbiomes (p = 0.05 and effect size = 0.1). The left side shows average relative abundance of metabolic features based on gut microbiome abundance and the right part visualizes the difference in mean proportions between the groups for each feature. Purple indicates the colic group; green is non-colic group. (**C**) (left) Ordination diagram of the variables of importance based on the random forest method. Shows metabolic pathways that are the best discriminators between the colic and non-colic groups. The horizontal axis is the measured effect of the variable on discrimination between groups across 30 permutation tests. Pathway names ranked by importance (↑) are shown on the vertical axis. (right) The ROC curve of the random forest analysis based on scores obtained in the leave-one-out validation for the entire 1 MB dataset. Only the top 20 significantly different functional features were used as predictors. The AUC value is the area under the corresponding curve. When the AUC is > 0.5 and the AUC value is closer to 1, the discriminative effect of the important variables is stronger.

To determine the metabolic changes in bacterial populations caused by the shift in bacterial abundance in the group with the colic phenotype, shotgun sequencing data were annotated in the MetaCys database using the HUMAnN 3 program^16^. The subsequent analysis using STAMP 2.1.3 software (p = 0.05 and effect size = 0.1) revealed 47 significantly different metabolic pathways (Fig. 3B).

As Fig. 3B shows, the metabolic pathways involved in glycolysis underwent the greatest changes, while in the group without colic, the pathways involved in amino acid biosynthesis had the highest relative abundance. The microbial community in the control (non-colic) group of infants was distinguished by the enrichment of the biosynthetic pathways for essential and non-essential amino acids, including branched-chain amino acids biosynthesis. Inosine-5-phosphate biosynthesis was increased in the non-colic group while its degradation was increased in the colic group. Ordination of metabolic pathways (Fig. 3A) using PCA in colic and non-colic groups did not demonstrate significant discrimination between them (ANOSIM = (R2 = − 1%, p = 0.5176), PEMANOVA = (F = 2.9, p = 0.1254). At the same time, subsequent random forest analysis based on the most differentially abundant pathways showed that changes in pathways between groups exhibited relatively high discriminative power, as quantified by a receiver operating characteristic (ROC) area under the curve (AUC) of 0.67 (see Fig. 3C(right)). Changes in metabolic pathways that have the highest significance (↑) for discriminating between colic and non-colic groups are shown in Fig. 3C(left).

**Figure 4:**
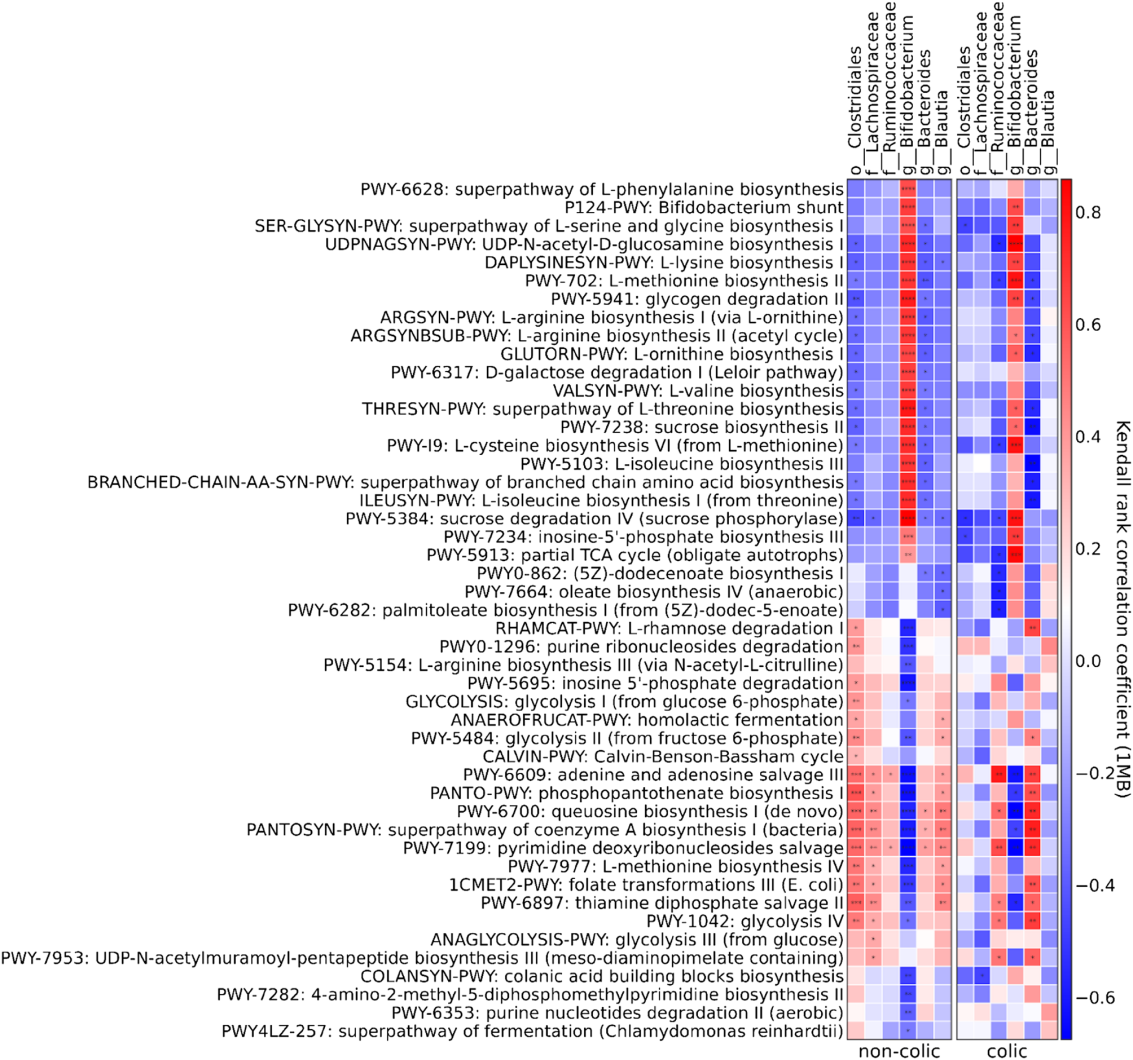
Correlation between significantly different taxa and metabolic pathways. Kendall rank correlation coefficient. Positive correlations are shown in red, and negative ones are in blue. p ≤ 0.05 highlighted in the center. Analysis points 1 month of life.

To understand the relationship between different taxa in the structure of microbial communities and metabolic activity, we performed a correlation analysis between these two groups. As Fig. 4 shows, *Bifidobacterium* genus in the non-colic group positively correlated with amino acid biosynthesis (l-lysine, l-threonine, l-methionine, l-isoleucine, l-serine), *Bifidobacterium* shunt, but negatively correlated with glycolysis, carbohydrate degradation, folate transformation. Whereas the order *Clostridiales* showed the opposite direction. *Bacteroides* genus was also negatively correlated with amino acid and *N*-acetyl-d-glucosamine biosynthesis. Whereas in the colic group, *Bacteroides* genus showed a high positive correlation with l-rhamnose degradation pathways, folate transformations, and glycolysis and negatively correlated with phospholipid biosynthesis and amino acid biosynthesis. *Blautia* genus in the colic group did not correlate with the most significantly different metabolic pathways.

## Discussion

In this study, we report identifying features of the colic gut microbiome in a cohort of 62 infants. We found that the starter set of microbiota obtained at birth makes a significant contribution to the formation of the colic phenotype. Recent studies have shown that the gut microbiome of infants with infantile colic is different from that of infants without colic. The key differences in microbial taxa between the two groups include lower levels of beneficial bacteria such as *Bifidobacterium, Lactobacillus*, and *Faecalibacterium prausnitzii*. These bacteria are known to play important roles in maintaining gut health, including the production of short-chain fatty acids (SCFAs) and the modulation of the immune system. Higher levels of opportunistic bacteria: infants with colic have been found to have higher levels of opportunistic bacteria such as *Clostridium, Escherichia coli*, and *Enterobacteriaceae*. These bacteria have been associated with gut inflammation, diarrhea, and other digestive issues. In the group of children with colic, a more rapid increase in the phylogenetic diversity of bacteria was revealed, which may indicate an accelerated maturation of the microbiome in these children. These dynamics continue until the cessation of colic; a snapshot of the microbiome at 12 months of age shows a relative leveling of biodiversity. This may indirectly indicate the influence of early intestinal microbial colonizers on the occurrence of infantile colic. It is also possible that the mode of nutrition has made an indirect contribution to the formation of the colic microbiome phenotype. The diet of children in the colic group up to 6 months of age consisted exclusively of breast milk without the addition of complementary foods or breast milk substitutes. The taxonomic structure of the microbiota of the children of the colic group at the 1 MB collection point was characterized by excessive diversity (Fig. 1A) with the predominance of the following taxa: *Bacteroides, Prevotella*, while *Bifidobacterium*, in contrast, showed a decrease in relative abundance, which, relative to the control group, remained reduced until 6 months of age (6 MB). Also, the linear discriminant analysis carried out additionally made it possible to identify taxa belonging to the class Clostridia, the relative number of which was increased on average in the group after the onset of colic. The microbiome enriched with these taxa, in our opinion, may be involved in the colic phenotype through the production of “intestinal gases”. Thus, it was found that *Bacteroides* and *Clostridium* produce H_2_, and CO_2_, as by-products of anaerobic fermentation, and, as a result, the accumulation of gases can be a source of discomfort for infants. A study of 40 infants found that the accumulation of lactate and hydrogen in the intestines of infants was associated with colic symptoms^7^. In addition, we found an increase in the relative abundance of the taxon *Ruminococcociae* (Fig. 2D), which was associated with the occurrence of behavioral problems in children aged 18–36 months^9^. From other studies, it can be traced that taxa related to *Bacteroidetes*, and *Clostridia* enrich the intestines of formula-fed children^18,19^. But, here we reported that the vast majority of children were breastfed and accordingly the relative abundance of these taxa was related to another factor at play. Such a factor may be maternal antibiotics in the perinatal period^20^. The investigation of the antibiotic factor in the future onset of colicky phenotype could be a good idea for future research, especially when considering antibiotic use by the mother during the last trimester. The meconium “starter microbiome” of children's intestines (3 DB microbiome snapshot) showed a significant difference in the relative content of dominant taxa. Thus, the composition of the “starter microbiome” of children with colic abounded in the taxa of *Bacteroides* and *Escherichia* and contained taxa of *Bifidobacterium, Enterococcus*, and *Klebsiella* to a lesser extent in comparison with the microbiome of the group of children without colic (Fig. 1A). The meconium microbiome could make some contribution to the microbiome development trajectory in the future. So in the meconium of the children of the colic group, before the onset of colic, the content of bifidobacteria was depleted, while the flowering of *Bacteroides* was increased. Mother's microbiota also plays an important role in the formation of an infant microbiome. Here we report the relationship between maternal enterotypes and infantile colic. We determined that 14 mothers (66.7%) from the children of the non-colic group had enterotype 2 (*Ruminococcus*) Supplement Table 1. Intergroup measurement of the similarity (β-diversity) of the diversity of intestinal microbiota samples of children with and without the colic phenotype confirmed the significance of the difference in the microbiota in the two groups of the study group cohorts (Fig. 2A, B). These differences are also explained by a significant decrease in the colic group of bifidobacteria and an increase in *Clostridia* and *Bacteroides* (Fig. 2D). We are continuing to monitor these groups of infants and collecting samples annually (up to adolescence), which we believe will shed light on the functional trajectory of microbiome development in children with infantile colic.

In order to comprehensively understand the differences between study groups, we analyzed the metabolic functions of microbial communities based on the MetaCys curatorial database. Clustering of metabolic pathways by study groups in PCA analysis (Fig. 3A) showed no significant differences, while analysis of extended error bar plots (Fig. 3B) revealed 47 significantly different metabolic pathways associated with taxonomic disturbance in the microbial community. Thus, we found that in the colic group the metabolic pathways involved in the biosynthesis of both essential (l-lysine, l-threonine, l-methionine, l-isoleucine, l-ornithine, l-valine, l-phenylalanine), and non -essential (l-serine, l-cysteine), semi -essential (l-arginine) amino acids were depleted, whereas the metabolic pathways involved in glycolysis reactions were enriched. Similar results were obtained in a study on a Chinese group of children born naturally^21^. Glycolysis (the Embden-Meyerhof-Parnass pathway) is known to be the most common pathway used by bacteria to produce H_2_^22^_,_ the excess accumulation of which can cause discomfort in infants. ROC analysis (Fig. 3C) of the most differentially abundant pathways between the microbiomes of children with and without infantile colic showed relatively high degree of discrimination. The correlation analysis between significantly different bacterial taxa and metabolic pathways in the colic and non-colic groups showed that genus *Bifidobacterium* had a significant positive correlation with the metabolic pathways involved in amino acid biosynthesis and negatively correlated with the relative abundance of nucleotide and nucleoside metabolism. In addition, the genus *Bifidobacterium* was negatively correlated with glycolysis in contrast to genus *Bacteroides*. In the colic group, as shown in Fig. 4, genus *Bacteroides* correlated with L-rhamnose degradation by nucleotide and nucleoside biosynthesis to a greater extent than in the non-colic group. The *Ruminococcaceae* family also had a stronger correlation in the colic group with the metabolic pathway of glycolysis, which may cause infant discomfort due to gas production as a by-product. It also correlated with the metabolism of oleic acid, which is known to contain 34–40% of all monounsaturated fatty acids (MUFAs) in breast milk^23,24^, the degradation of which also results in the accumulation of intestinal gas^25,26^. *Blautia* genus, although significantly increased in the colic group, did not show significant correlations with significantly different metabolic pathways, but has been associated with BMI and obesity in other reports^27^.

### Limitations

The conducted study and the interpretation of its results have certain limitations. In the course of recruiting, we recruited 62 healthy newborns, of which, due to the inclusion criteria in the study, the size of the groups was significantly reduced. To understand the composition of the microbiota of breastfed children with infantile colic, we limited the sample according to the mode of nutrition. Since participation in the study was voluntary and participants could opt-out at any stage, this also had a significant impact on the sample size. Also during nucleic acid extraction, it turned out that not all meconium samples contained insufficient amounts of bacterial DNA, which also reduced the sample size. Thus, our study group consisted of only 15 children with confirmed colic and 21 control groups. However, high dropout rates did not affect the statistically significant measurable effects.

## Conclusions

It is important to note that the gut microbiome of infants is complex and dynamic and that many factors may contribute to colic symptoms. The meconium microbiome and its formation can influence the future developmental trajectory of the microbiota and influence positive or negative outcomes through biosynthesis, useful organic molecules, carbohydrate degradation, gas release. The relationship between the gut microbiome and colic is still not fully understood, and further research is needed to identify specific microorganisms or microbial pathways that are associated with colic and to determine how these microorganisms interact with other environmental and genetic factors that may contribute to colic symptoms.

## Supplementary Information

Supplementary Figure 1.

Supplementary Table 1.

## Data Availability

The datasets presented in this study can be found in online repositories. The names of the repository/repositories and accession number(s) can be found below: NCBI BioProject [accession number PRJNA949528].
