# Supplementary material for: Compositional and functional variability of the gut microbiome in children with infantile colic
Source: Sci Rep. 2023 Jun 12;13:9530. doi: 10.1038/s41598-023-36641-z (PMC10261102; doi:10.1038/s41598-023-36641-z)
Supplement: Supplementary file 1 — Supplementary Figure 1. [file 41598_2023_36641_MOESM1_ESM.pdf]

Supplement figure 1. Alpha biodiversity indices

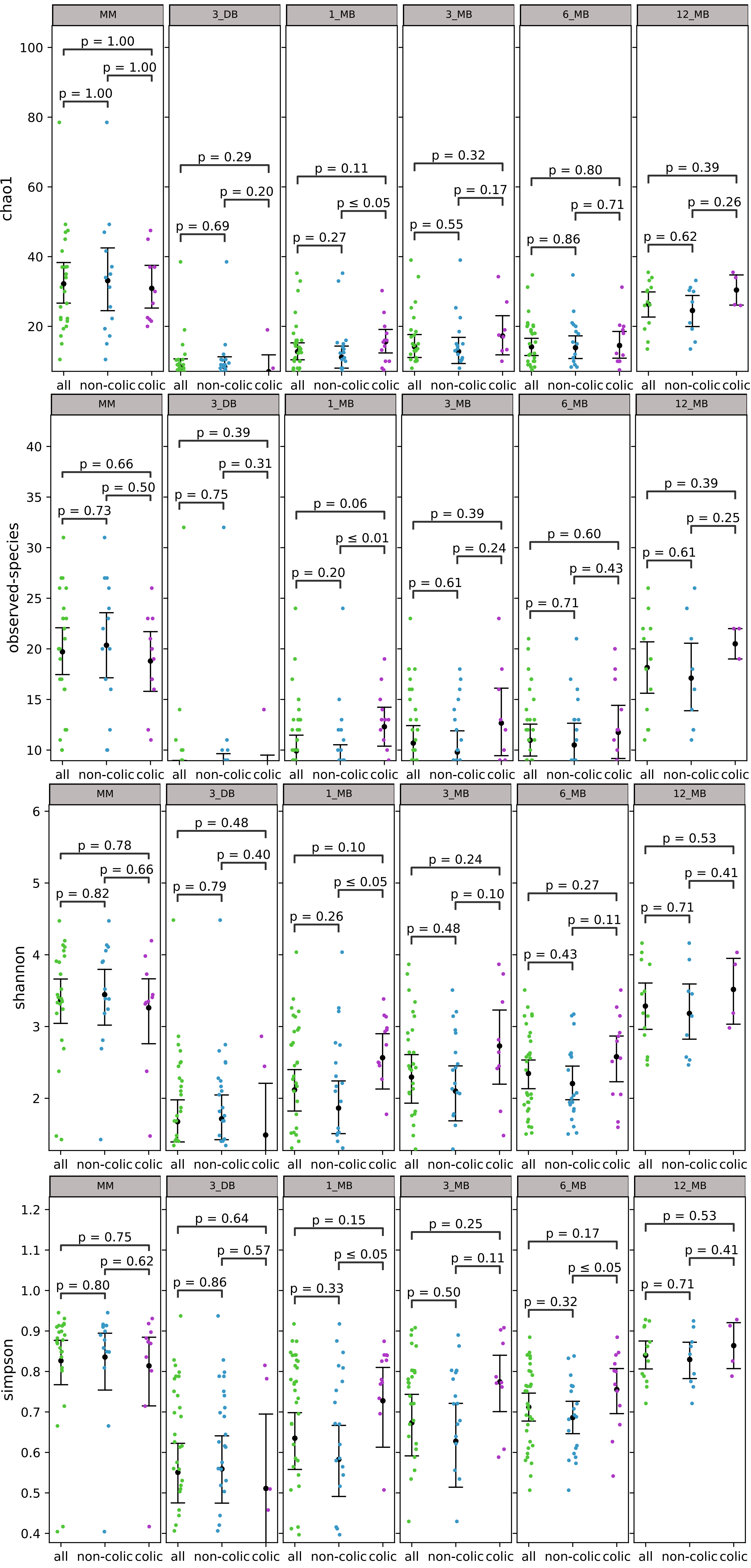

Alpha biodiversity (Chao1, Observed, Shannon, Simpson indices at the species level) in maternal (1 month after childbirth) and infant samples across the entire cohort and rates with and without infantile colic after birth and up to 12 months of life; red dots - all groups; green dots - non-colic group; purple dots - colic group, whereas the whiskers represent the 95% confidence interval (CI) (with 2000 bootstrap resampling); Mann-Whitney U test.
